# Flame-Retardant Polyamide Powder for Laser Sintering: Powder Characterization, Processing Behavior and Component Properties

**DOI:** 10.3390/polym12081697

**Published:** 2020-07-29

**Authors:** Kevin Schneider, Katrin Wudy, Dietmar Drummer

**Affiliations:** 1 Institute of Polymer Technology, Friedrich-Alexander-Universität Erlangen-Nürnberg, Am Weichselgarten 9, 91058 Erlangen, Germany; dietmar.drummer@fau.de; 2 Professorship of laser-based Additive Manufacturing, Department of Mechanical Engineering, Technical University of Munich, Boltzmanstraße 15, 85748 Garching b. Munich, Germany; Katrin.wudy@tum.de

**Keywords:** selective laser sintering, polyamide 12, flame-retardant polymer, flammability

## Abstract

Up to now, laser-sintered components have been barely used by industries such as aerospace and transport industry due to high flammability. By the use of flame retardants, the flammability of laser-sintered parts should be reduced to extend their range of possible applications. This paper aims to investigate the influence of halogen-free phosphinate-based flame retardants on process-relevant characteristics and process behavior, as well as mechanical and physical properties. Most importantly, the flammability of the material should be reduced. Two different types of phosphinate-based fillers were used in a concentration between 10 and 25 wt % in combination with the matrix material polyamide 12 (PA12). Thermal, optical, and powder properties of the mixtures were analytically investigated. Furthermore, the mechanical characterization of the sintered specimen was carried out. The addition of filler in laser sintering changes the process behavior and properties of the component. With this investigation, the correlation among flame retardants, process-relevant characteristics, process behavior, and resulting part properties was derived for the first time. Finally, a mixture of 15–20 wt % of flame retardant leads to the best trade-off between flame retardancy and mechanical properties.

## 1. Introduction

Laser sintering (LS), a laser-based powder bed fusion process, is widely spread in prototype construction virtually in all branches of industry. However, this technology has only been able to assert itself for the production of series components in a few cases. One example is the individualization of the side scuttles and cockpit facia at MINI Yours Customized, where MINI owners can customize their car. However, for critical applications such as aerospace and transportation, laser-sintered components usually do not meet flame retardancy requirements because of the generally high flammability of plastics. This effect is further amplified by the high surface area of laser-sintered components. In plastics processing, polymer materials are usually filled with flame retardants to achieve an increased flame retardancy. At the same time, the used organic fillers inevitably lead to a changed process behavior in laser sintering. Therefore, the aim of the investigations was to analyze the influence of halogen-free phosphinate-based flame retardants on the process behavior and the resulting properties of laser-sintered components. Finally, the best trade-off among flame retardancy, mechanical properties of the laser-sintered components, and processing behavior in laser sintering should be reached with a specific mixture.

## 2. State of the Art and Theoretical Consideration

### 2.1. Laser Sintering of Polymers

The principle of laser sintering is to selectively heat and fuse plastic microparticles by the use of a CO_2_ laser. The building process consists of three phases: preheating, building, and cooling. The aim of the preheating phase is to create a homogeneous starting layer and temperature conditions. During the building phase, the three process steps material coating, energy input, and material consolidation are repeated until the components are built up. With a roller or knife, the bulk material (average particle size approximately 50 µm) is applied in the building chamber, whereby the layer thickness is set between 80 and 150 µm. According to the theory of quasi-isothermal laser sintering of semi-crystalline polymers, the building chamber is preheated to a temperature between the onset of melting and crystallization [1,2]. The energy to fuse the polymer particles selectively is introduced via a CO_2_ laser. During phase change from enthalpy elastic state to viscous flow, the particles begin to coalesce, resulting in a homogeneous molten layer. According to the Frenkel model [3], particle coalescence is mainly driven by viscosity and surface tension of the melt. The surrounding powder particles act as supporting structures for the molten layers and remain loosely in the building chamber. The lowering of the building chamber by the thickness of one layer is followed by the coating process and the energy input. These three process steps are repeated for each layer until all cross-sections of the components have been generated. The surrounding powder and the components are cooled after the building phase until the melt solidified [4].

### 2.2. Materials in Laser Sintering

In principle, any plastic material that is fusible under heat can be processed in laser sintering. For the production of mechanically loaded components, mainly semi-crystalline materials are used. Commercially available semi-crystalline materials are, for example, polyamide 12 (PA12), polyamide 11 (PA11), and polyetherketone (PEK) [5,6]. Besides the mentioned polymers, thermoplastic elastomers and some amorphous thermoplastics are commercialized [6]. In the past, huge efforts to develop new polymer powders for laser sintering, such as polybutylene terephthalate (PBT) [7,8,9], were made. However, the market is dominated by PA12, PA11, and filled grades of these polymers [5,6]. A possible reason for the slow market introduction of new laser sintering polymer powders is the low market share with 900 t/year compared to the worldwide plastic use of 260 million t/year [6].

Despite the huge efforts in material development, available materials cannot fully meet the need of different functional end-use parts. In particular, if components are to exhibit flame-retardant behavior, the corresponding materials are missing. This behavior is due to the chemistry of polymers, which mainly consist of carbon and hydrogen. Plastics, in general, show high fuel values. For example, the energy values of polypropylene (PP) (43 kJ/g) and polyethylene (PE) (41.6 kJ/g) [10] are almost as high as those of heating oil (45.4 kJ/g) [11]. To reduce the energy released during the combustion, flame retardants can be added to the plastics. Thereby, the aim of a flame retardant is to elongate the time until a polymer material catches fire and at the same time reduce the heat released of the burning polymer to a minimum [12,13]. Depending on the purpose of the components, various flame retardants, differing in their chemical structures, are available. In addition, other fillers such as glass spheres, glass fibers, carbon fibers, or talcum can reduce the combustibility of a plastic material by dilution of the flammable material.

Hitherto existing research activities in the field of composite materials in laser sintering focus on the improvement of mechanical properties by adding microscopic inorganic fillers such as glass spheres [14,15], silicon carbide [16], aluminum [17] or nanoscaled additives [18,19]. Only a few studies on the influence of additives on fire behavior for laser-sintered parts have been published.

Koo et al. [20] analyzed the influence of nanoparticles on flammability, thermal, and mechanical behavior of PA12 and PA11. Koo et al. [20] produced various mixtures of chemically modified montmorillonite (MMT) organoclays, surface modified nanosilica, and carbon nanofibers (CNFs) with twin-screw extrusion. The injection-molded specimen were analyzed according to their flammability and mechanical performance [20]. Based on the highest flame retardancy, some mixtures were cryogenically ground to microsized powder and processed in laser sintering [20]. Nevertheless, the flammability properties for the mixtures are only a little enhanced compared to the raw PA12 and PA11 material.

Lao et al. melt-blended PA11 and PA12 with low concentrations of nanoparticles, namely nanoclays, carbon nanofibers, nanosilicas, and flame retardant additives, to enhance the thermal properties and analyzed the thermal, flammability, and mechanical properties, as well as the morphology [21,22]. They manufactured the composite material via injection molding to accelerate the material development step but did not conduct any laser sintering trials. Therefore, the process behavior in the aimed technology laser sintering is not validated for these materials.

### 2.3. Flame Retardant Additives and Mechanism

Flame retardants can be differentiated according to the type of reaction under the influence of heat. On the one hand, physical additives act by diluting the combustible polymer in solid and gaseous phases. They can also decompose in an endothermic reaction, which leads to energy consumption [23,24]. On the other hand, chemically reacting fillers influence the chemical processes of polymer decomposition. They can either react with radicals generated during the combustion process, which usually causes an acceleration of the material decomposition, or they can lead to charring of the top layer of the sample [23,25,26,27]. A highly promising type of reaction is the intumescence [12,24,27]. It describes a combination of charring and inflating of the sample’s top layer causing a separation of flame and burnable polymer.

Composite materials consisting of functional fillers, such as flame retardants and polymers, will show a different process behavior in laser sintering compared to pure polymers. Nevertheless, the addition of flame retardants is indispensable for generating individualized parts with specific flammability, which is required for applications in the aerospace or transport industry. Therefore, the fundamental process behavior of composite materials, namely flame retardants and polymers, and resulting component properties were investigated in this study. For the investigations, a physical mixture of flame retardant and PA12 powder was used. The behavior of a particle-filled system would probably lead to different thermal, optical, and rheological material behaviors.

## 3. Experimental Methodology

### 3.1. Materials

#### 3.1.1. Polymer

A PA12 powder type PA2200 from the supplier EOS GmbH (Krailling, Germany) was used for the investigations. This semi-crystalline thermoplastic powder has been developed for laser sintering. In addition to a high bulk density (0.44 g/cm³) and a low viscosity (viscosity number 60 mL/g according to ISO 307, solvent: sulfuric acid, virgin powder), the material has a pronounced hysteresis between the beginning of the melting (peak maximum of the first heating 185 °C) and crystallization (peak maximum of the crystallization 147–150 °C). The volumetric median particle size of the PA12 powder is 63 µm. A refresh rate of 50% virgin and 50% used powder was used as the material supplier’s recommendation.

#### 3.1.2. Flame Retardants

As flame retardants, the two halogen-free, phosphinate-based fillers Exolit OP 1230 and Exolit OP 1400 from Clariant AG (Frankfurt am Main, Germany) were used. The median particle size of the material Exolit 1230 is between 20 and 40 µm and of the material Exolit 1400 around 35 µm. Thus, both flame retardants show a particle size near the PA12 powder (d50 63 µm), whereby good miscibility is expected. The smaller the particles are, the higher the van der Waals forces become, which may result in aggregation and worse flowability. This kind of additives achieves a flame retardant effect by building up a char layer at the surface of the component in case of fire. Thus, the atmospheric oxygen and the combustible polymer are separated. At the same time, the phosphorous components of the flame retardant act as radical reactors in the gaseous phase by removing highly reactive H– and OH– radicals in the burning zone. These radicals usually have a huge influence on the decomposition of polymers. As described by the manufacturer, both types of flame retardants used are optimized for polyamide materials such as PA6 or PA12. The manufacturer gives a phosphorus content of 23.5–26% for Exolit OP 1400 and 23.3–24% for Exolit OP 1230.

#### 3.1.3. Composite Materials

To enhance the flame retardancy of the investigated material PA12, the two types of flame retardants were added separately. In these investigations, a physical powder mixture of flame retardant and the plastic powder was investigated. Resulting material behavior is only valid for this mixture. If the flame retardant were melt-mixed and thus distributed in the particles, the behavior would be different. A rotary mixer from Somakon Verfahrenstechnik UG type MP 25 (Lünen, Germany) was used for the homogenization of the physical mixture. The rotation speed was set to 400 r/min for a mixing time of 20 min. Due to the expected changes of laser-powder interaction and necessity of a high flame retardant content, the filler content was varied between 12 and 25 wt %. The nomenclature of the material mixtures is displayed in Table 1.

### 3.2. Laser Sintering

#### 3.2.1. Processing Conditions

Laser sintering experiments were carried out at a commercial LS system Formiga P110 from the supplier EOS GmbH (Germany). The systems building space is 200 × 250 × 320 mm³. The powder coating was realized with a rake mounted on a rotating spindle. The system was equipped with a CO_2_ laser with a maximum laser power of 30 W. The building chamber was heated by an infra-red (IR)-radiator above the powder bed surface. The withdrawal chamber was heated separately.

The use of fillers or additives leads to a change in the process behavior of the composite materials. For example, the thermal behavior of filled and un-filled materials differs [28]. Therefore, a temperature search was carried out based on the recommended building temperature of the matrix material. Therefore, cross-shaped specimens were evenly distributed over the building space to detect curling effects. If a curling effect occurs at the set building chamber temperature, the temperature is gradually increased. Table 2 shows the resulting building chamber temperatures for the used materials mixtures.

The machine parameters (Table 3) scan speed $v_{s}$, hatch distance $h_{s}$, and layer thickness were kept constant, while the laser powder $P_{L}$ was changed in order to achieve different energy densities at equal interaction time. The energy density can be calculated according to equation 1.

(1) $$E_{D}=\frac{P_{L}}{v_{s}\cdot h_{s}}$$

#### 3.2.2. Specimen Geometry and Process Layout

Three different types of specimen were built in one building process. The density of the components was measured on cubes with an edge length of 20 mm. For Limiting Oxygen Index (LOI) and UL-94 tests, specimens with a length of 120 mm, a width of 10 mm, and a thickness of 1 mm were produced. Tensile test bars according to DIN EN ISO 527-2 type 1A were built for mechanical testing.

**Figure 1:**
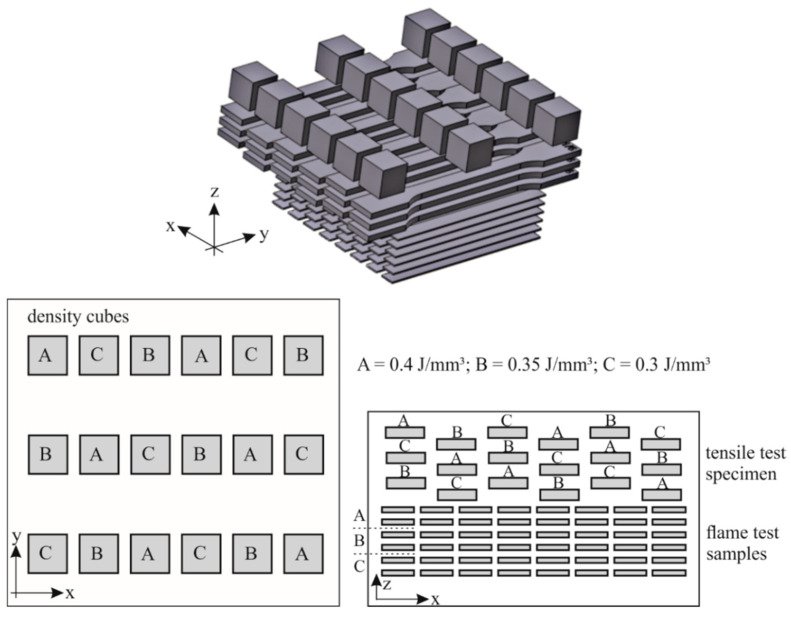
Building process layout and classification of the energy density of the cubes for density measurements.

For each mixture, the building layout illustrated in Figure 1 was used. In the first step, the test specimens for the LOI and UL-94 tests were produced. Two layers with eight samples each were prepared for each energy density (Table 3). Subsequently, tensile test specimens were manufactured in the XY direction with varying energy densities. In total, three levels of tensile test specimen were built, resulting in a total of 18 specimens. For each energy density, five tensile test specimens were used for mechanical testing while the sixth was used for microscopic analysis. Finally, 18 cubes for density analysis were built with statistically distributed energy density.

### 3.3. Material Characterization

#### 3.3.1. Scanning Electron Microscopy (SEM)

The investigation of particle size and shape of the used flame retardant agents was carried out on a scanning electron microscope (SEM) from Carl Zeiss AG (Oberkochen, Germany). Additionally, the fractured surfaces of the mechanically tested samples with filler concentrations of 0, 12, and 20 wt % Exolit 1230 were analyzed. All images were taken at a working distance of 10 mm and an electron gun voltage of 5 kV. For the investigation of the flame retardant particles, a magnification of 1000× was used. In the case of the analysis of the fractured surfaces, a magnification of 20× was selected to create an overview image. Detailed images were taken with a magnification of 1000×. For all images, the secondary electron detector was used.

#### 3.3.2. Bulk Density

Besides thermal properties, powder characteristics are the key properties for processing in laser sintering. The bulk density of the polymer powder and the composite materials was determined according to DIN EN ISO 60. The bulk density is defined as the weight of a bulk consisting of particles divided by their occupied volume. For the measurements, a system from Emmeram Karg Industrietechnik (Krailling, Germany) type ADP was used. Based on the bulk density, the porosity of the bulk $\varepsilon_{\mathrm{bulk}}$ was calculated with the following equation:

(2) $$\varepsilon_{\mathrm{bulk}}=1-\frac{\rho_{\mathrm{bulk}}}{\rho_{\mathrm{solid}}}$$

where the bulk density is $\rho_{\mathrm{bulk}}$ and the density of a solid material is $\rho_{\mathrm{solid}}$.

For the composite materials, the density was related to the composition of the materials and the equation for calculating the bulk porosity changed to:

(3) $$\varepsilon_{\mathrm{bulk}}=1-\frac{\rho_{\mathrm{bulk}}}{\rho_{\mathrm{solid},m}\cdot \left(1+\varphi_{f}\right)+\rho_{\mathrm{solid},f}\cdot \varphi_{f}}$$

where the density of the polymer matrix material is $\rho_{\mathrm{solid},m}$, the density of the filler is $\rho_{\mathrm{solid},f}$, and the gravimetric filler concentration is $\varphi_{f}$.

The analysis of the bulk porosity is necessary to compare the bulk characteristics of the different composite materials and to analyze whether the space between the particles is changed as a function of filler content.

#### 3.3.3. Diffuse Reflectance Infrared Fourier Transformation Spectroscopy (DRIFTS)

With diffuse reflectance infrared Fourier transformation spectroscopy (DRIFTS), the infrared spectra of powders can be determined with minimum sample preparation. In DRIFTS, electromagnetic radiation reflected from dull surfaces is collected and analyzed as a function of frequency (ν, usually in wavenumbers, cm^−1^) or wavelength (λ, usually in nanometers, nm). For the analysis, an infrared spectrometer Nicolet 6700 (Thermo Scientific, Waltham, MA, USA) with a DRIFTS unit Diffus IR (PIKE Technologies, Fitchburg, WI, USA) was used.

#### 3.3.4. Differential Scanning Calorimetry (DSC)

The thermal material properties were analyzed by differential scanning calorimetry (DSC). A system from TA Instruments (New Castle, DE, USA) type Q 2000 was used. The scan rates for heating and cooling were set to 10 K/min while the specimens’ weight was 5 mg. Besides the non-isothermal DSC experiments, an isothermal crystallization analysis was performed. Therefore, the specimens were heated up to a temperature of 200 °C with a heating rate of 10 K/min and cooled down to the crystallization temperature of 162 °C or rather 166 °C by a cooling rate of 60 K/min. The heat flow caused by exothermal crystallization was measured as a function of time.

#### 3.3.5. Heat Capacity

The material’s heat capacity may have a decisive influence on its resulting temperature during the laser sintering process, as the developed heat is linearly linked to the heat capacity. Therefore, the heat capacity was determined with a C80 calorimeter from the manufacturer Setaram Instrumentation SAS (Caluire-et-Cuire, France). The measurements were started at a temperature of 18 °C and stopped after reaching 30 °C. The heating rate was set to 0.1 K/min. A mass between 4000 and 7000 mg was used for the measurements. The value of the heat capacity was determined at 23 °C.

#### 3.3.6. Thermogravimetric Analysis (TGA)

Thermogravimetric analysis (TGA) is a thermal characterization method in which the change of the weight of a sample is measured over temperature or time. For the plastic powder and the composite, the degradation behavior can be investigated. For the measurements, a system from TA Instruments (Germany) type Q5000 was used. The heating rate was set to 10 K/min and the sample mass was approximately 5 mg.

#### 3.3.7. Flame Tests

To investigate the fire behavior of the composite, the two widely used tests, LOI and UL-94, were carried out. For both tests, a sample geometry of 120 × 10 × 1 mm³ was used. In LOI, the minimum amount of oxygen in the atmosphere required for a steady burning of the specimen is measured. Therefore, a sample is placed vertically in a testing tube while it is fixed on the bottom side. The sample is then exposed to a constant gas flow of a predefined mixture of nitrogen and oxygen and ignited with a defined flame at the top side for a maximum of 30 s. As soon as the sample is burning independently under this defined atmosphere, the ignition flame is removed. Then, the time is determined until the sample extinguishes by itself or a measuring mark is reached, which is set 50 mm below the sample’s top edge. The test is repeated with a new sample and a lower oxygen percentage of the testing atmosphere in the case the flame reached the marker or a higher oxygen level if the specimen was self-extinguished. The oxygen level is increased by 0.2% if the sample extinguishes before the flame reached the marker and the burning time is shorter than 3 min. The oxygen level is lowered by 0.2% if the flame or burning melt of the plastic reaches the marker and for burning times longer than 3 min. In an iterative testing procedure according to DIN EN ISO 4589-2, the oxygen concentration between constant burning and self-extinguishing is detected, which is defined as the Oxygen Index (OI). A higher OI value indicates a better flame retardancy of the material.

According to DIN EN 60695-11-10, the UL-94 test can be carried out with either vertical or horizontal sample orientation. For the vertical setup, the specimen is fixed at the top edge. A defined flame is placed at the bottom edge for 10 s. After the removal of the ignition flame, the time until the self-extinguishing is measured. This procedure is repeated once. The samples are classified according to the sum of burning times, dripping behavior, or burning up to the clamp. Possible ratings in descending order are V-0, V-1, and V-2. If none of the ratings is achieved, the sample can be tested in the horizontal test to get the classification HB. Therefore, the sample is ignited from one side for 30 s. To pass the test, the burning rate has to be beneath a defined value or the sample has to extinguish by itself. Compared to the LOI, the UL-94 test is strongly operator dependent. That is why both techniques were compared in this investigation.

#### 3.3.8. Density

Due to the specimen production out of a powder material at ambient pressure, the density of the resulting parts may be influenced by the energy density and the filler content. To determine the density of the different materials, cubes with an edge length of 20 × 20 × 20 mm³ were produced. The weight of the components was measured by a fine balance of the manufacturer Pescale Waegetechnik GbmH (Bisingen, Germany) with a resolution of 1 µg. The density of one cube was then calculated by dividing the measured weight by its volume.

#### 3.3.9. Mechanical Tests

To analyze the influence of filler on the mechanical properties mechanical tests were performed. To compare the mechanical properties of the different materials, the samples were dried in a vacuum oven at 70 °C up to constant weight conditions. All tests were performed on a universal testing machine type 1464 from the manufacturer Zwick GmbH & Co. KG (Ulm, Germany) according to DIN EN ISO 527-1 under standard climate at 23 °C and relative humidity of 50%. For each material, five samples were tested. For the determination of the modulus of elasticity, the tensile test was initiated at a speed of 1 mm/min in the beginning. It was then accelerated to 50 mm/min for the investigation of the maximum tensile strength and elongation at break.

#### 3.3.10. Optical Microscopy

The optical microscopy was used to characterize the filler distribution as well as the pore form and amount within the samples. Cross-sections with a thickness of 10 µm were prepared from tensile test bars and density cubes to analyze the influence of energy density and filler content on the resulting distribution of the fillers and pores. Therefore, both bright field and polarized light modes were used. All images were taken on an Axio Imager.M2m (Carl Zeiss AG, Oberkochen, Germany) with a magnification factor of 25×.

## 4. Results and Discussion

### 4.1. Influence of Flame Retardants on Bulk Characteristics

**Figure 2:**
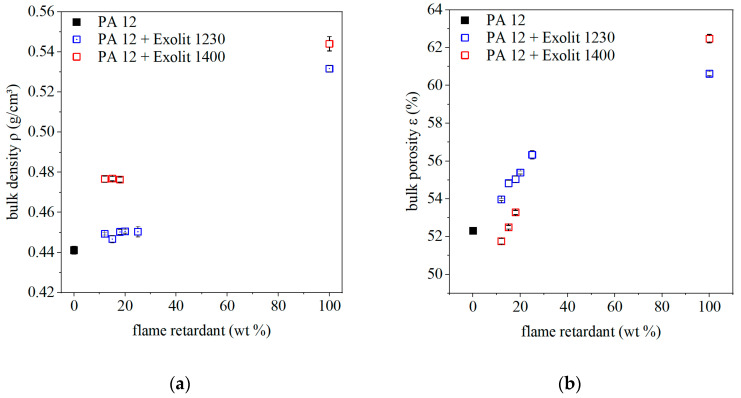
Bulk density (**a**); and bulk porosity (**b**) of the polymer and composite powders for different flame retardant concentrations.

A homogeneous powder bed surface and a high powder bed density is prerequisite for the generation of an even molten layer in laser sintering. Therefore, the used powder must have high flowability and bulk density. The bulk density of the composite powders consisting of PA12 and different flame retardants with varying filler concentrations is represented in Figure 2. With increasing filler content, the bulk density increases due to a higher density of the flame retardant. The density of the flame retardant type Exolit OP 1230 is 1.35 g/cm³, and, for Exolit OP 1400, it is 1.40 g/cm³. Due to the almost same size and shape of both types of additives, a higher bulk density for the type Exolit OP 1400 results.

**Figure 3:**
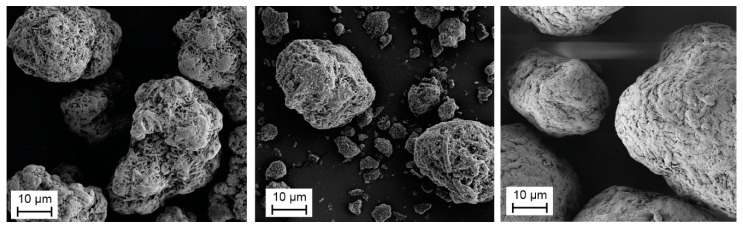
SEM images of the flame retardant fillers Exolit 1230 (**left**) and Exolit 1400 (**middle**) and the PA12 powder (**right**) with a magnification factor of 1000×.

For a direct comparison of the two flame retardants concerning their process behavior, the analysis of the bulk density is not applicable, due to differing density. Therefore, the bulk porosity is calculated based on density and bulk density according to Equation (3). With increasing flame retardant content, the bulk porosity increases. The SEM images in Figure 3 show that the median particle size of the flame retardant Exolit 1230 is between 20 and 40 µm. The powder shows a homogeneous distribution of particle size. The flame retardant Exolit 1400 has almost the same median particle size as Exolit 1230 with a d_50_ value of 35 µm, but a broader distribution, as visualized in Figure 3. This broader particle size distribution of Exolit 1400 compared to Exolit 1230 in combination with the PA12 powder results in reduced bulk porosity. The smaller particles are able to fill the space between the PA12 particles and thus the bulk porosity is reduced. These different porosities occur due to the physical mixture of the powder. If the flame retardant could be distributed in the particles itself the bulk porosity should stay constant as long as the particle size distribution is equal.

A smaller bulk porosity will lead to less trapped air during the melting of the polymer powder and finally to a higher density of the specimen. However, this assumption only applies if the melting of the powder is not altered by the fillers. Melting is determined by the energy input, which can be changed by the addition of fillers due to a changed absorption behavior.

### 4.2. Influence of Flame-Retardants on Optical Properties

**Figure 4:**
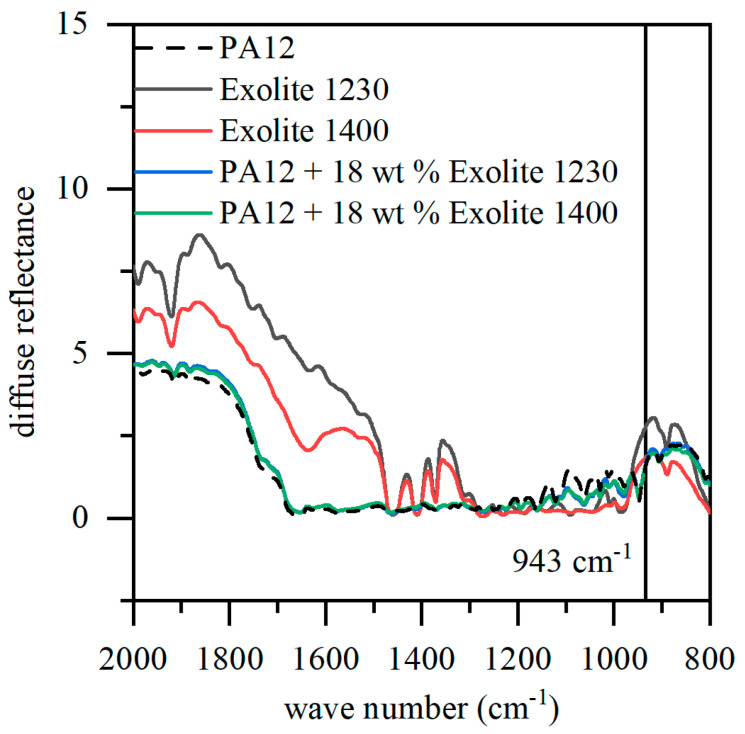
Diffuse reflectance infrared spectra of filler, PA12 powder, and composite powders.

The optical properties of the powder determine the necessary energy input in laser sintering and may change by the addition of fillers. The analysis of optical properties of powders differ from the optical properties of solid material due to rough surface and thus diffuse reflected beams. DRIFT spectra of the pure material (fillers and polymer powder) as well as of the mixtures are represented in Figure 4. PA12 powder (broken line) shows a low diffuse reflectance and thus a high absorption at the wavelength of the CO_2_ laser (943 cm^−1^). Both flame retardants have a slightly higher diffuse reflectance at this wavelength. Nevertheless, a high and almost same absorption can be detected for both mixtures of PA12 and Exolit. Thus, the mixtures should be manufactured with similar energy densities typical for PA12 powder. If, for example, the diffuse reflectance of the filled powder were higher compared to the plastic powder, the total energy input would have to be increased.

### 4.3. Influence of Flame-Retardants on Thermal Properties

**Figure 5:**
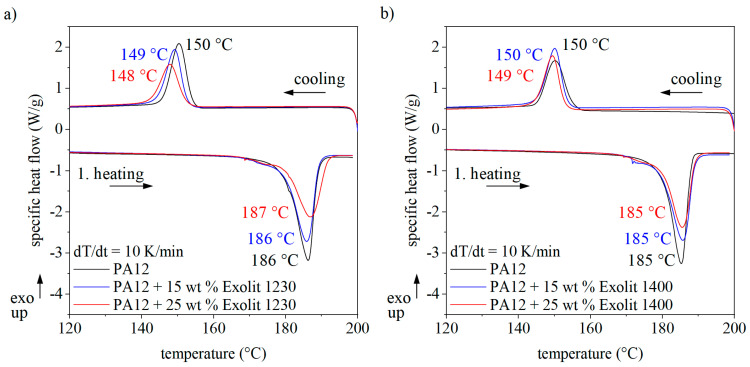
Thermogram of the polymer and composite powders for different flame retardant concentrations.

Besides the bulk characteristics and the optical properties, the thermal behavior under processing conditions of the composite powder is essential for successful processing in laser sintering. Two different DSC analyzing modes, isothermal and dynamic measurements, are well-established to evaluate the thermal behavior in laser sintering. With dynamic measurements, the building chamber temperature can be estimated. The first heating and cooling of PA12 powder and two representative composite powders are shown in Figure 5. With increased flame retardant content, the melting temperature does not change, but the enthalpy is reduced with increased flame retardant content due to a lower amount of plastic material that can melt and crystallize. The onset and the peak maximum of the crystallization decrease slightly with rising flame retardant content. Thus, the additive does not act as an initial nucleus. Nevertheless, the reduced crystallization temperature will lead to a changed processing behavior and the theoretical processing window.

**Figure 6:**
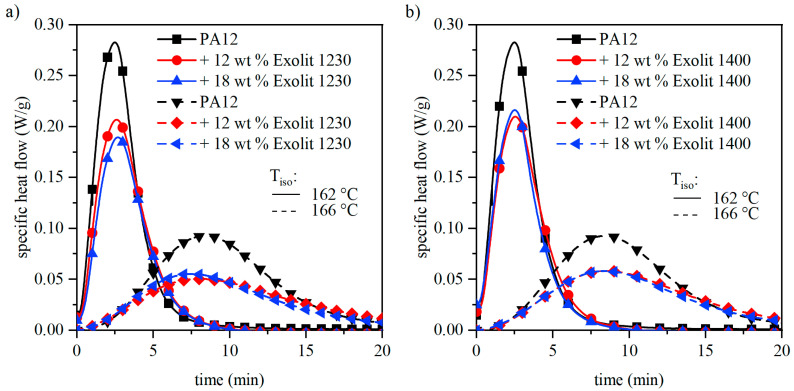
Isothermal DSC measurements of the polymer and composite powders for different flame retardant concentrations.

To analyze the effect of the filler more closely, isothermal DSC measurements were carried out. The temperature for isothermal measurements was chosen according to the process chamber temperature of 166 °C and at a lower value to accelerate the crystallization process. The corresponding heat flow curves for pure PA12 and the composite powders are shown in Figure 6. The area below the curve represents the proportion of a material able to crystallize and is thus reduced for composite powders. For isothermal measurements, the time at which the heat flow curve reaches the peak maximum is of particular interest. This time is independent of the flame retardant content, which indicates a stable processing behavior similar to pure PA12 powder.

Figure 5 and Figure 6 prove that the crystallization of the composite powder is not influenced by the flame retardants. Nevertheless, the thermal household in laser sintering could differ between plastic powders and composite materials due to changed heat capacity. Table 4 represents the measured heat capacities for PA12 and the composite powders. The addition of flame retardant decreases the material’s heat capacity. This is caused by the lower capability of the flame retardant powder to store heat. The energy input in a system can be calculated according to the following equation:

(4) $$∆Q=c_{p}\cdot m\cdot ∆T$$

where ∆Q is the change of energy in a system, $c_{p}$ is the heat capacity, m is the mass, and ∆T is the temperature change.

The thermal household in laser sintering is complex. While the energy input to fuse the powder is applied by the CO_2_-laser, the temperature of the applied powder layer is managed by (IR) radiators. Crucial in laser sintering is the curling effect, which is reflected in the formation of the warpage in the topmost layer. The curling effect indicates an unbalanced temperature deviation between the topmost and the previously exposed layer and is particularly critical for filled systems. According to Keller [29], thermal stresses arise between two layers due to temperature differences, which may lead to the so-called curling effect. The temperature difference is determined by the building chamber temperature and thus the powder temperature and the temperature after laser exposure. If the thermal stresses exceed a critical value without the possibility to relax in the given time, the building processes are interrupted. For pure PA12 powder, building chamber temperature and matching laser parameters are well investigated. However, for filled systems, the thermal properties heat capacity and thermal conductivity are changed. If the building chamber temperature and the exposure parameter for PA12 are set for filled systems, the varying thermal properties will result in differing temperature distribution. During laser exposure, a fixed laser parameter set will lead to the same energy input but a higher temperature difference due to the lower heat capacity of the composite material. This higher temperature difference may indicate a first curling effect. To avoid curling, two strategies are possible. One the one hand, the laser power and energy input can be decreased, to reduce the temperature difference and thus curling. On the other hand, the building chamber temperature can be raised, and the temperature difference will be reduced. Choosing the first option the plastic material may not melt completely, resulting in a bad interconnection of the particles. Therefore, the building chamber temperature was increased in this investigation.

### 4.4. Degradation Behavior of the Composite Powder and Flame Retardancy of the Laser-Sintered Specimen

**Figure 7:**
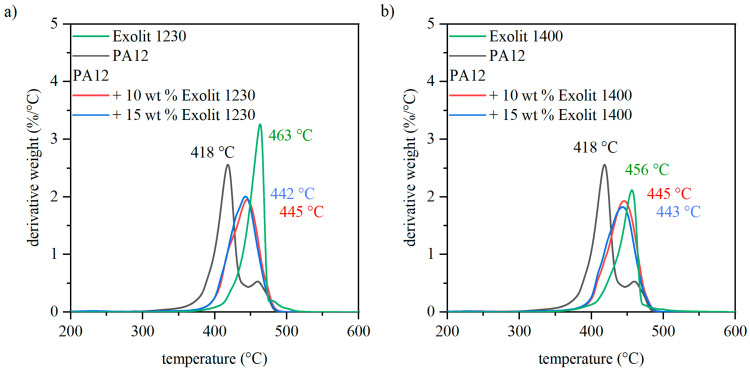
Derivative weight over the temperature of the polymer, flame-retardant filler, and composite powders for different flame retardant concentrations.

The degradation behavior of the composite powders under defined conditions was measured by thermogravimetric analysis. Therefore, the powder specimens were heated to a temperature of 800 °C with a heating rate of 10 K/min. The reduction of mass depending on the temperature is displayed as the first derivative of the weight loss curve in Figure 7. The evaluation of the first derivative of the weight loss curve was selected because this makes it much easier to analyze whether one or more maxima occur at the speed of the weight loss. The highest mass loss of the PA12 powder occurs at lower temperatures compared to the flame retardant and composite powder. Additionally, the degradation behavior of both fillers is slightly different. Exolit 1400 (Figure 7 right) shows a maximum at the first derivative of the weight loss curve at 456 °C, whereas the maximum is reached at 463 °C for the flame retardant Exolit 1230. Nevertheless, the mixtures of plastic and flame retardant indicate that the flame retardant has a similar effect on the degradation behavior for both flame retardants. The peak of the first derivative of weight loss for composites with both fillers is clearly shifted to higher temperatures. However, the degradation temperature does not follow a linear mixing rule, since the maximum temperatures are closer to pure fillers, although their proportion is only 1015 wt %. Small amounts of flame retardant lead to a large effect on the degradation behavior of the powder. Nevertheless, this effect cannot be directly transferred to the flame behavior of laser-sintered specimens due to complex fire behavior.

**Figure 8:**
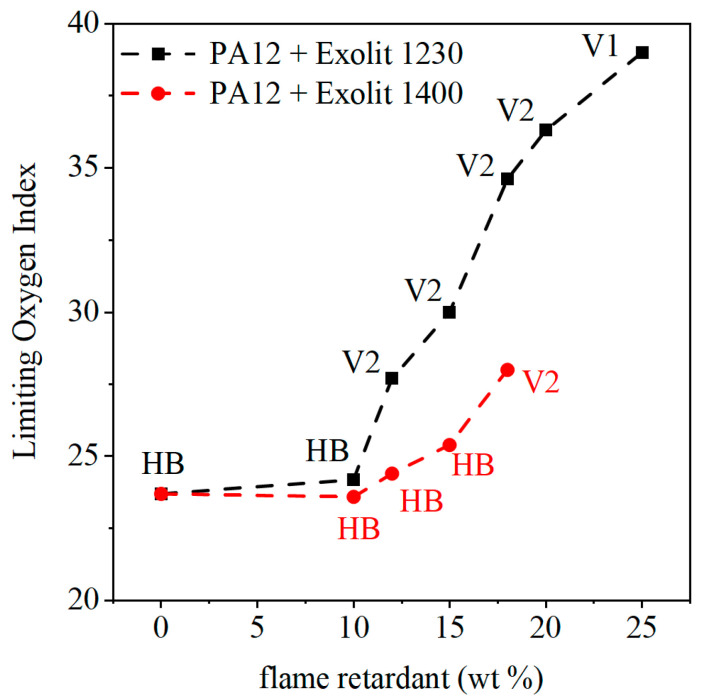
LOI and UL-94 results of the laser-sintered specimen for different flame retardant concentrations.

The fire behavior of the laser-sintered specimen was analyzed by LOI and UL-94 test. The LOI of pure PA12 and composite specimens is shown in Figure 8. For low filler contents, the LOI remains on a constant level of 24% independent of the used flame retardant. For filler grades above the threshold value of 10 wt %, an increase in the LOI was achieved. This behavior may be caused due to a lower plastic content as well as the rising effect of the flame retardant. With the flame retardant Exolit 1230, higher LOI values can be reached compared to Exolit 1400. Hence, the flame retardant Exolit 1230 exhibits better fire behavior with the same flame retardant content. Increasing filler contents lead to a more brittle material behavior, which is represented by changing mechanical behavior which is crucial in laser sintering.

The UL-94 levels of the 1 mm specimen are also displayed in Figure 8. Unmodified PA12 specimens show an inadmissible fire behavior for applications in aerospace or electrical engineering with a UL-94 rating of HB. A concentration of 10 wt % flame retardant does not lead to an improvement in fire behavior. As displayed in Figure 8 an LOI of 24% in combination with a UL-94 rating of HB could be measured for pure PA12 and mixtures with 10 wt % of flame retardant. With increasing flame retardant, the fire behavior can be changed to a UL-94 rating of V-1 for the flame retardant Exolit 1230 with a concentration of 25 wt %. The measured LOI for this specimen is 39%. All investigated flame retardant concentrations between 10 and 25 wt % achieved a V-2 rating with constant increasing LOI values. The classification V-1 implies that the summed up burning time per sample does not exceed 30 s while at least one specimen shows a combined burning time for both inflammations between 10 and 30 s. The aimed UL-94 rating would be V-0. To reach this rating, all specimens must burn less than 10 s without flaming droplets. Flaming droplets automatically lead to a V-2 rating for burning times up to 30 s. The UL-94 test is failed if one of the five specimens burns for more than 30 s or if the flame reaches the clamping. The laser-sintered specimens with 25 wt % Exolit 1230 show an inhomogeneous fire behavior. Three specimens would be rated with V-0 and the other two with V-1. This could be traced back to inhomogeneous density and local flame retardant concentrations. Therefore, microscopic images of the tensile test specimen were analyzed. With the use of Exolit 1400, the achievable LOI values and UL-ratings are worse for the same amount of flame retardant.

**Figure 9:**
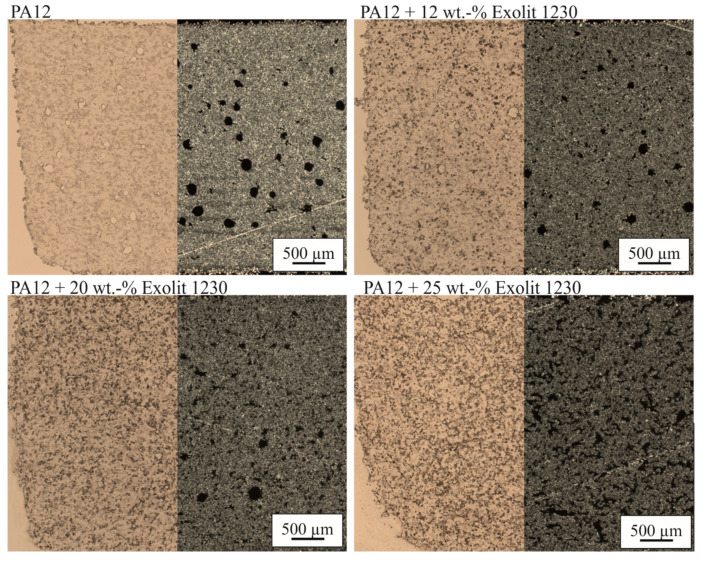
Microscopy images (bright field and polarized light) of the laser-sintered tensile test specimen for different flame retardant concentrations and an energy density of 0.35 J/mm³.

Figure 9 illustrates microscopic images in both bright field and polarized light of laser-sintered tensile test specimens of PA12 filled with Exolit 1230. Pores within the specimen can be displayed by polarized light (right side of the images), whereas with bright-field images (left side of the images) filler distribution can be visualized. In all specimens, the flame retardant, which is visible as black dots in bright field, is homogeneously distributed. The grey areas in the PA12 specimen show the silicon dioxide-based flow agent. With increasing flame retardant, the form and amount of pores change. For low filler concentration, spherical pores occur. The shape of the pores converts to irregular channels with increasing filler content. The higher porosity in combination with the increased flame retardant content leads to a superposition of flame retardancy and thus to the reached UL-94 rating of V-1 and LOI of 39% for the specimen with an Exolit content of 25 wt %.

### 4.5. Physical and Mechanical Properties of the Laser-Sintered Specimen

**Figure 10:**
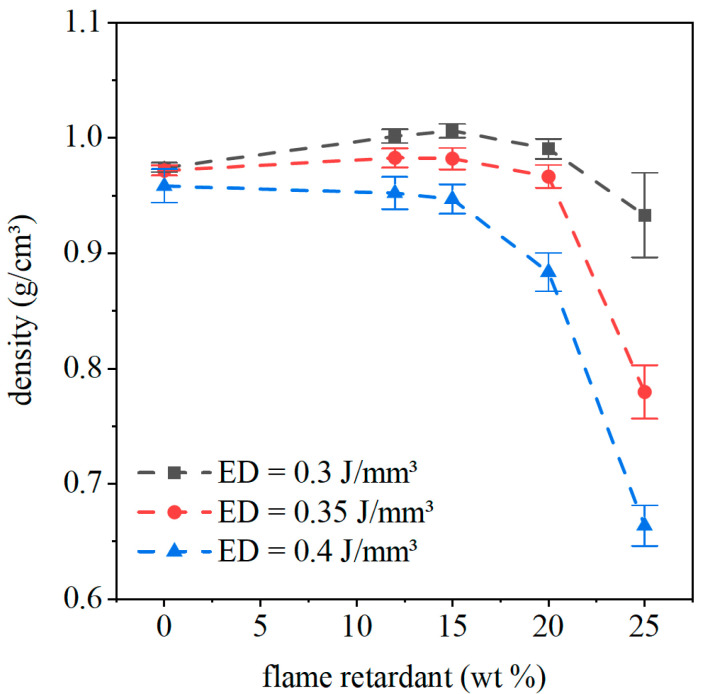
The density of the laser-sintered specimen for different flame retardant concentrations and energy densities.

With the flame retardant Exolit 1230, better fire behavior can be reached. Therefore, mechanical tests and density analyses were only performed for specimens filled with Exolit 1230. The density of laser-sintered cubes with different flame retardant concentrations and energy densities are represented in Figure 10. There is no significant influence of the energy density on the density of the specimen visible for PA12. In comparison, the applied energy density for filled systems shows an influence on the part density. The density of the part decreases constantly with increasing energy density, which is mostly caused by adhesion of powder particles on the surface. These particles adhere due to the changed heat conductivity of the composite material. Up to a flame retardant concentration of 15 wt %, the part density for all specimen manufactured with the same energy density stays constant. For higher concentrations, the density decreases. This effect can be visualized by the microscopy images in Figure 9 and Figure 10. The amount of pores is increased for specimens with a flame retardant concentration of 20 and 25 wt %.

**Figure 11:**
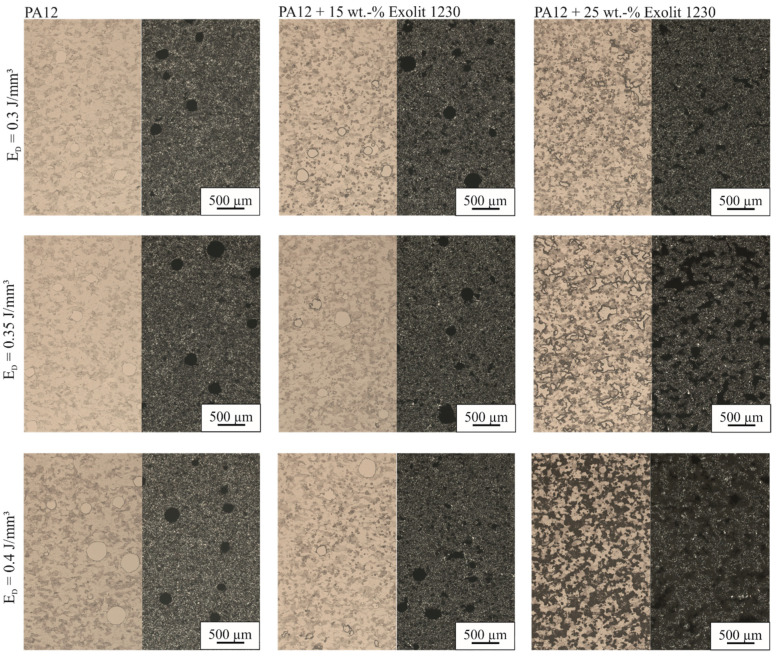
Microscopy images (bright field and polarized light) of the laser-sintered density cubes for different flame retardant concentrations and energy densities.

Images of laser-sintered thin sections of the density cubes are represented in Figure 11. To work out the influence of flame retardant content and energy density on filler distribution and amount as well as dimension and form of the pores, images were taken in bright field and polarized light. Under polarized light, the pores are visible, whereas in bright field the flame retardant can be seen. With increasing flame retardant content, the pore shape changes from spherical pores for PA12 to an irregular shape. The pores become smaller, but the number of pores increases. With increasing energy density, the pore size and form do not change, which goes along with the density results. For an Exolit 1230 concentration of 15 wt %, the pore shape changes with increasing energy density from spherical to irregular pores. This threshold can be detected by density measurements in Figure 10 as well. The density of the cubes decreases with increasing energy density beginning at a flame retardant level of 15 wt %. For higher flame retardant contents, this effect is enlarged. For the highest energy density (0.4 J/mm³) in combination with the highest flame retardant concentration (25 wt % Exolit 1230), the pores cannot be separated from the Exolit powder, while there is no connection between the PA12 matrix visible. A higher energy density leads to a higher energy input into the powder for constant exposure times (i.e., constant scan speed). Due to the higher thermal conductivity of the material depending on the Exolit concentration, an increased amount of energy flows through the top layer into deeper layers. This results in enhanced heating of the underlying material whereby the amount of energy used for melting processes is reduced. Therefore, the number of pores rises with increasing flame retardant content. Additionally, the amount of adhered powder particles on the surface of the specimen rises. The combination of adhering powder particles on the cube surface and the rising amount of pores in the specimen results in low densities for high flame retardant concentrations and high energy densities. Due to the sharp drop of density and missing specimen accuracy, specimens with high Exolit 1230 contents should not be used as construction materials.

**Figure 12:**
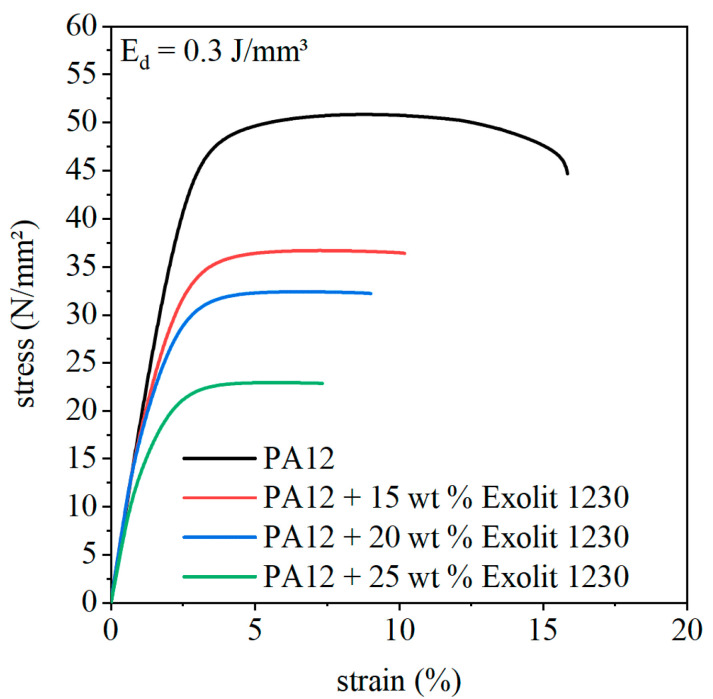
Averaged stress–strain curves of laser-sintered specimens for different flame retardant concentrations and an energy density of 0.3 J/mm³.

Typical stress–strain curves of a laser sintered PA12 specimen are shown in Figure 12. Laser sintered specimens do not have a distinct tensile yield point followed by necking and drawing. Nevertheless, mechanical behavior can be classified as ductile without a tensile yield point. Averaged stress–strain cures of the composite materials for an energy density of 0.3 J/mm³ are also represented in Figure 12. With increasing flame retardant concentration, the fracture behavior changes from ductile to brittle failure. This goes along with a reduction of elongation at break and a decrease of maximum tensile strength. The same trends can be seen for 0.35 and 0.4 J/mm³ energy density.

**Figure 13:**
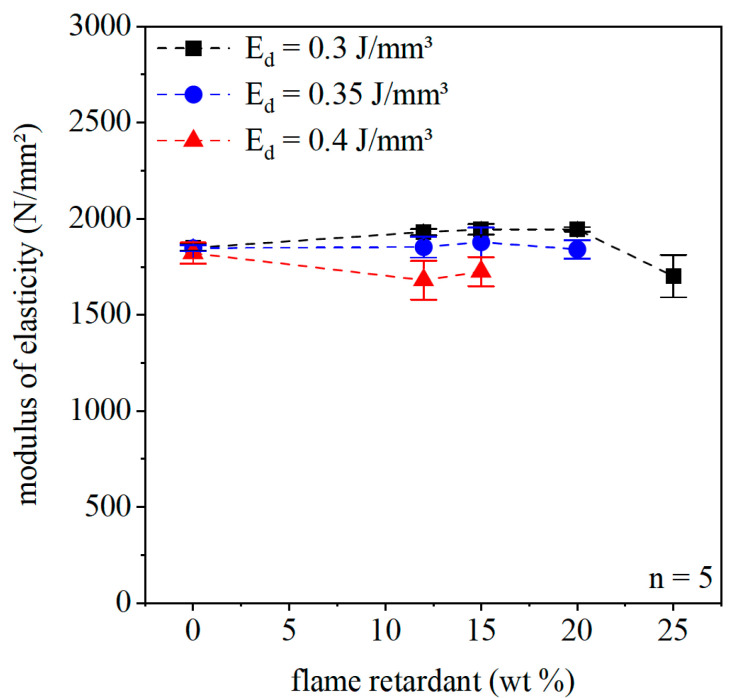
Modulus of elasticity of the laser-sintered specimen for different flame retardant concentrations and energy densities.

Modulus of elasticity is an important parameter to characterize the stiffness of the material, and it is used as one key construction parameters. Furthermore, the change of the modulus of elasticity indicates the interfacial bonding between the polymer matrix and fillers. Figure 13 illustrates the effect of flame retardant on the modulus of elasticity for the three energy densities. Similar trends can be observed for all applied energy densities. The elastic modulus does not change with increasing flame retardant content up to 20 wt % of Exolit 1230. For a filler content of 25 wt %, the elastic modulus drops slightly. Typically, organic fillers have a reinforcing effect. In [30], the elastic modulus of an injection molded PP composite with a halogen-free and halogenated flame retardant rises with increasing filler content due to the higher modulus of the fillers. Unfortunately, this reinforcement effect cannot be detected here, which is a hint for an insufficient interfacial bonding between the matrix and the flame retardant. In [21], Lao et al. improved the mechanical properties of laser-sintered PA11 and PA12 specimens with flame retardant contents between 20 and 25 wt % by adding nanoparticles, namely nanoclays (NCs), carbon nanofibers (CNFs), and nanosilicas (NSs). These nanoparticles act as reinforcement additives. Nevertheless, the composite of PA12 in combination with flame retardant shows the same effect of constant elastic modulus levels or a slight drop of elastic modulus, as described in this paper. To improve the bonding between the flame retardant and the PA12 matrix, a modification of the filler surface could be helpful for further investigation.

**Figure 14:**
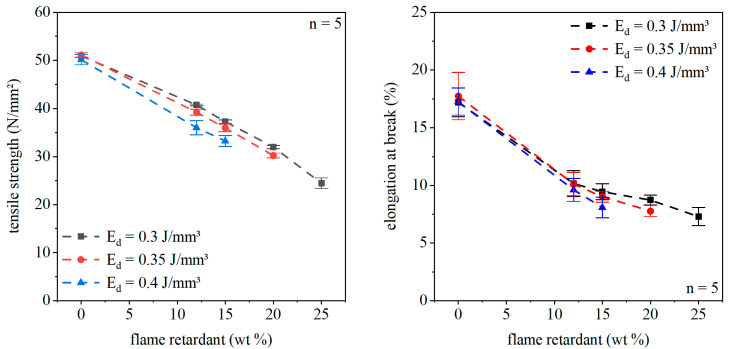
Tensile strength and elongation at break of the laser-sintered specimen for different flame retardant concentrations and energy densities.

Figure 14 displays the dependency between tensile strength and flame retardant concentration for varying energy density. Due to missing accuracy in shape, the specimens with a high flame retardant content and high energy densities cannot be examined. For low flame retardant content, the specimen failed in a ductile manner. This behavior changes to a brittle fracture with increasing filler content. The tensile strength decreases with increasing flame retardant concentration and rising energy input.

The reduction of tensile strength with increasing flame retardant concentration indicates an insufficient bonding between polymer matrix and filler or a fracture of the flame retardant particles. For higher energy densities this effect is enhanced by a growth of the specimen size due to loosely adhered powder particles. The so-adhering particles are only partially melted, leading to a formation of sinter necks without continuous interconnection. This is caused by the enhanced heat conductivity and the simultaneously lowered heat capacity. Forces cannot be transferred by these structures. Therefore, the adhesion of particles results in an additional decrease in calculated strength. Whether the interparticle bonding or the bonding between filler particles and the matrix material is the weak spot of the investigated specimen was analyzed by SEM images of the fracture surface of the tensile test samples.

**Figure 15:**
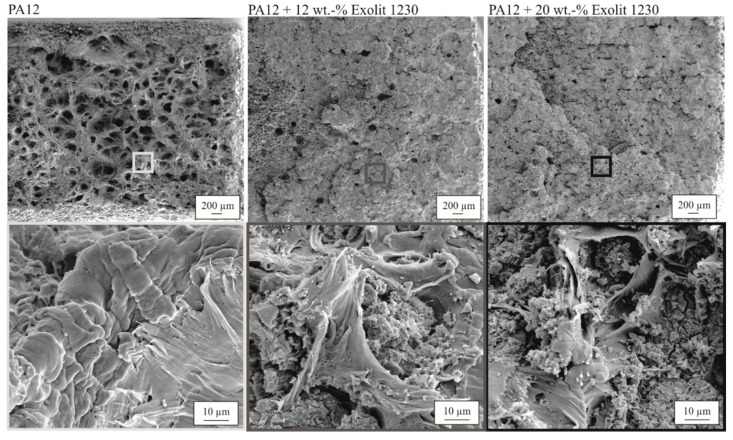
Fracture surface after tensile testing for specimens with different flame retardant concentrations and an energy density of 0.35 J/mm³.

The SEM images in Figure 15 show sufficient bonding between filler particles and matrix material. The filler particles are embedded in the matrix material after fracture and no gap between the flame retardant and PA12 materials is visible. Compared to the SEM image of the flame retardant in Figure 3, the particles seem to be torn apart, which is a hint for a weak interparticle bonding. Thus, it can be assumed that the connection between matrix material and filler is quite good as no holes remain in the fracture area from ripped out filler particles. The brittle fracture surface results from the internal breakage of the filler material. This assumption is supported by the cubic particles on the surface. The relatively weak interparticle bonding or the weak bonding between the flame retardant particles and the PA12 matrix is superposed by the raising amount of pores. Both influences lead to a drop in tensile strength.

In laser sintering, the elongation at break is a primary parameter, which reacts very sensitively to changes in the material. The elongation at break depending on flame retardant concentration and energy density is represented in Figure 14. With rising filler content, the elongation at break decreases. Again, the drop in elongation at break is due to increasing pore number and rising filler content. The flame retardant particles and the pores act as a potential defect, which can initiate a crack.

With the SEM images in Figure 15, the previously presented results for the mechanical measurements can be additionally confirmed. As shown in the overview pictures, the ductile behavior of pure PA12, from which the frayed structures emerge, is changed to a smooth or plate-like fracture surface, which is an indicator of brittle material behavior.

## 5. Conclusions

Up to now, laser-sintered components do not interfuse with industries such as aerospace or transportation due to inadequate flame retardant properties of these components. Pure plastics usually burn very well. The addition of flame retardants improves the flame retardancy of plastics. Nevertheless, additional fillers change the process behavior in laser sintering and resulting part properties. The process behavior of filled plastics with flame retardant was investigated in both analytical and process-related approaches. Furthermore, resulting flame retardancy and mechanical properties, which are crucial in laser sintering, were investigated.

Mixtures of Exolit 1230 and Exolit 1400, two halogen-free, phosphinate-based flame retardants with concentrations between 10 and 25 wt % (10, 12, 15, 18, 20, and 25 wt %), were used in combination with PA12 as matrix material. Powder flowability, powder porosity, and thermal and optical properties are key characteristics for successful laser sintering trials. The powder porosity increases with increasing filler content. This effect is more severe for the flame retardant Exolit 1230 due to a homogeneous particle size distribution. The broader distribution of the filler Exolit 1400 leads to a higher density because of the filling of gaps with small particles. The crystallization behavior of the PA12 matrix material is not changed by the investigated flame retardants. This was verified by non-isothermal and isothermal DSC measurements. Nevertheless, the overall thermal household in laser sintering is modified by the addition of filler. This can be exemplified by curling occurring for the composite materials, which can be traced back to reduced heat capacity, leading to a rise of building chamber temperature. Thus, not only the crystallization behavior but also the capability to store heat is a crucial aspect in laser sintering and should be taken into account.

The flame retardancy was investigated on laser sintered specimens with different flame retardant concentrations. These specimens were produced with different energy densities. Unfortunately, the flame retardant Exolit 1400 leads only to a slight increase in fire behavior and was not chosen for further mechanical investigations. Exolit 1230 shows a better flame retardancy with a UL-94 rating of V-1 for a concentration of 25 wt %. The specimens with 12, 15, 18 and 20 wt % exhibit a UL-94 rating of V-2. Despite the same UL-94 rating, the LOI is further increased for the Exolit 1230 with increasing flame retardant concentration. Analysis of the density of the samples shows it growing with increasing filler content and energy density due to reduced heat capacity and enlarged thermal conductivity. Concurrently, the porosity of the specimen increases with the filler content. Both effects are superposed and lead to a reduction of tensile strength and elongation at break for rising flame retardant content. The slight reduction of modulus of elasticity is furthermore a hint for an insufficient bonding between the particles and the matrix or a weak interparticle bonding. SEM analysis of the fracture surfaces reveals a weak interparticle bonding and a tearing of filler particles. An energy density of 0.35 J/mm³ in combination with a flame retardant concentration of 15–20 wt % leads to parts with appropriate mechanical and flame retardancy properties.

## Figures and Tables

**Table 1:** Powder mixtures.

Composition/Sample Name	Content Exolit 1230 in (wt %)	Content Exolit 1400 in (wt %)
PA12 + 10 wt % FR (flame retardant)	10	10
PA12 + 12 wt % FR	12	12
PA12 + 15 wt % FR	15	15
PA12 + 18 wt % FR	18	18
PA12 + 20 wt % FR	20	-
PA12 + 25 wt % FR	25	-

**Table 2:** Used building chamber temperature for the composite powders.

Filler Degree (%)	0	10–15	18–20	25
Building chamber temperature (°C)	168	174	176	178
Withdrawal chamber temperature (°C)	150	150	150	150

**Table 3:** LS system parameter.

Parameter-set	*E_D_* (J/mm³)	*P_L_* (W)	*v_s_* (mm/s)	*h_s_* (mm)	d (mm)
A	0.4	25	2500	0.25	0.1
B	0.35	22	2500	0.25	0.1
C	0.3	19	2500	0.25	0.1

**Table 4:** Heat capacity of the polymer and composite powders at 23 °C.

	Heat Capacity (J/g·K)		
Material composition	PA 2200	Exolit 1230	Exolit 1400
PA12	1.692	-	-
PA12 + 18 wt % flame retardant	-	1.611	1.592
pure flame retardant	-	1.336	1.268
